# Effect of Sec61 interaction with Mpd1 on endoplasmic reticulum-associated degradation

**DOI:** 10.1371/journal.pone.0211180

**Published:** 2019-01-25

**Authors:** Fabio Pereira, Mandy Rettel, Frank Stein, Mikhail M. Savitski, Ian Collinson, Karin Römisch

**Affiliations:** 1 Faculty of Natural Sciences and Technology, Saarland University, Saarbruecken, Germany; 2 Proteomics Core Facility, EMBL, Heidelberg, Germany; 3 School of Biochemistry, University of Bristol, Bristol, United Kingdom; Universite de Geneve, SWITZERLAND

## Abstract

Proteins that misfold in the endoplasmic reticulum (ER) are transported back to the cytosol for ER-associated degradation (ERAD). The Sec61 channel is one of the candidates for the retrograde transport conduit. Channel opening from the ER lumen must be triggered by ERAD factors and substrates. Here we aimed to identify new lumenal interaction partners of the Sec61 channel by chemical crosslinking and mass spectrometry. In addition to known Sec61 interactors we detected ERAD factors including Cue1, Ubc6, Ubc7, Asi3, and Mpd1. We show that the CPY* ERAD factor Mpd1 binds to the lumenal Sec61 hinge region. Deletion of the Mpd1 binding site reduced the interaction between both proteins and caused an ERAD defect specific for CPY* without affecting protein import into the ER or ERAD of other substrates. Our data suggest that Mpd1 binding to Sec61 is a prerequisite for CPY* ERAD and confirm a role of Sec61 in ERAD of misfolded secretory proteins.

## Introduction

In eukaryotes about 30% of all proteins constitute secretory pathway cargo [1]. These proteins are transported into the ER by the conserved heterotrimeric Sec61 channel formed by Sec61, Sbh1, and Sss1 in yeast (Sec61α, Sec61ß, Sec61*γ* in mammals) [2]. During ER targeting and translocation the Sec61 channel interacts with multiple other protein complexes on its cytosolic face and in the ER membrane including the ribosome, the SRP receptor, the Sec63 complex, oligosaccharyl transferase, and signal peptidase [3–7]. If proteins fail to fold in the ER, they trigger the unfolded protein response (UPR), unless they are transported back to the cytosol for ERAD [8,9]. Although this process has been intensely studied for over 20 years, the identity of the retrograde transport channel is still controversial. The first and most investigated candidate is the Sec61 channel [10]. More recently, its role in ERAD has been called into question, mainly because of two arguments: 1) A number of (mainly transmembrane) ERAD substrates were found to be "Sec61-independent". In all of these experiments, however, the weak, temperature-sensitive *sec61-2* allele was used to investigate ERAD at its permissive temperature such that Sec61 channel was active. Whenever restrictive conditions and stronger mutant alleles were used, *sec61* mutants were defective in ERAD (summarized in [10]). 2) Mutants in *SEC61* may lead to a reduced concentration of an essential ERAD factor in the ER, hence the effect of the *sec61* mutants on ERAD is indirect. The first mutant used to investigate the role of the Sec61 channel in ERAD, however, *sec61-32*, was cold-sensitive for ER import, but defective for ERAD at the permissive temperature for import [11]. Moreover, since then, two *sec61* mutants have been identified in lumenal loop 7 which are fully competent for protein import into the ER, but specifically defective in ERAD [12,13]. The conformation of lumenal loop7 affects binding of the proteasome 19S regulatory particle to the cytosolic face of the channel [13]. The 19S regulatory particle has been shown to extract a yeast ERAD substrate from the ER and is involved in ERAD of several substrates in mammalian cells (summarized in [10]). The proteasome 19S particle hence can serve as an alternative "extraction motor" to Cdc48 which can also bind to Sec61 [14]. Bacterial and plant toxins (Shiga, ricin, and cholera) which need to be transported from the ER to the cytosol also interact with the Sec61 channel and require its activity (summarized in [10]). All three toxins also need the E3 ubiquitin ligase Hrd1 for transport to the cytosol. Hrd1 and the pseudorhomboid proteases Der1 and Dfm1 have been proposed more recently as alternative ERAD channels [15,16]. The Sec61 channel has been shown to interact with Hrd1, and Hrd1 with Der1, so these proteins may also operate together in transporting ERAD substrates to the cytosol [17,18,10].

If the Sec61 channel is involved in retrograde transport of ERAD substrates, it would have to interact with ERAD factors targeting ERAD substrates to its lumenal end. While Sec61 interaction with ERAD substrates has been shown [11,19], the only known ER lumenal ERAD factor that is known to interact with Sec61 at its lumenal loop 7 is the Hsp70 BiP [20]. Here we have used chemical crosslinking and mass spectrometry to identify new interactors of Sec61 with specific focus on ERAD-relevant and lumenal interactors in order to better understand the role of the Sec61 channel in this process. We show that the PDI homolog and CPY*-specific ERAD factor Mpd1 binds to the ER-lumenal hinge region of Sec61 and that deletion of the Mpd1-binding site in Sec61 leads to an ERAD-defect specific for CPY*.

## Materials and methods

*S*. *cerevisiae* strains used in this study are listed in *Table 1*, plasmids in Table 2, primers in Table 3, and antibodies in Table 4.

**Table 1:** *S*. *cerevisiae* strains used in this study.

Name	Genotype	Reference
KRY37	*MATa his4 trp1 leu2 ura3 HOL1-1 sec61-3*	[21]
KRY157	*Mat*α *leu2 his3 trp1 ura3 ade2 sec61*::*HIS3 can1-100 [pDQ sec61-32]*	[11]
KRY160	*MATa leu2 his3 trp1 ura3 ade2 can1-100 leu2*::*LEU+UPRE-lacZ MET+ ire1*::*TRP1*	[22]
KRY461	*MAT*α *sec61*::*HIS3 leu2 trp1 prc1–1 his3 ura3 [pGAL- SEC61-URA3]*	[12]
KRY853	*MAT*α *leu2 ura3 [pRS306-truncsec61-S353C]*	[13]
KRY897	*MAT*α *sec61*::*HIS3 leu2 trp1 prc1–1 his3 ura3 [pRS315- SEC61-LEU]*	[12]
KRY1061	*MAT*α *sec61*::*HIS3 leu2 trp1 prc1–1 his3 ura3 [pRS315- pGal-14His-Sec61S353C-LEU]*	This work
KRY1081	*MAT*α *sec61*::*HIS3 leu2 trp1 prc1–1 his3 ura3 [pRS315- pGal-14His-SEC61-LEU]*	This work
KRY1116	*MAT*α *sec61*::*HIS3 leu2 trp1 prc1–1 his3 ura3 [pRS315- sec61del1-LEU]*	This work
KRY1117	*MAT*α *sec61*::*HIS3 leu2 trp1 prc1–1 his3 ura3 [pRS315- sec61del2-LEU]*	This work
KRY1118	*MAT*α *sec61*::*HIS3 leu2 trp1 prc1–1 his3 ura3 [pRS315- sec61del1/2-LEU]*	This work
KRY1162	*MAT*α *sec61*::*HIS3 leu2 trp1 prc1–1 his3 ura3 [pRS315- SEC61-LEU] [pRS426-MPD1-HA-URA]*	This work
KRY1163	*MAT*α *sec61*::*HIS3 leu2 trp1 prc1–1 his3 ura3 [pRS315- sec61del1-LEU] [pRS426-MPD1-HA-URA]*	This work
KRY1164	*MAT*α *sec61*::*HIS3 leu2 trp1 prc1–1 his3 ura3 [pRS315- sec61del2-LEU] [pRS426-MPD1-HA-URA]*	This work
KRY1165	*MAT*α *sec61*::*HIS3 leu2 trp1 prc1–1 his3 ura3 [pRS315- sec61del1/2-LEU] [pRS426-MPD1-HA-URA]*	This work

**Table 2:** Plasmids used in this study.

Name	Description	Reference
pBW11	*SEC61* in pRS315	[12]
pRS315	CEN vector (*LEU2*)	[23]
pRS426	2μ vector (*URA3*)	[24]
pRS315-*sec61S353C*	*sec61S353C* in pRS315	[13]
pRS315-His_14_-*sec61S353C*	*GAL1-His*_14_*-sec61S353C* in pRS315	This work
pRS315-His_14_-*SEC61*	*GAL1-His*_14_*-SEC61* in pRS315	This work
pRS315-*sec61del1*	*sec61del1* in pRS315	This work
pRS315-*sec61del2*	*sec61del1* in pRS315	This work
pRS315-*sec61del1/2*	*sec61del1/2* in pRS315	This work
pRS426GAL1	pGAL1+ N-terminal His_14_-tag	[25]
p416pΔgpαF	overexpression of pΔgpαF (*URA3*), contains MET25 promoter	[26]
pSM101	*KWW-HA* (*URA3*)	[27]
pSM70	*KHN-HA* (*URA3*)	[27]
pYM24	hphNT1 marker with 3xHA tag	[28]

**Table 3:** Primers used in this study.

Name	Sequence (5’-3’)	Restriction Site	Application
Primer 1	ATGTCCTCCAACCGTGT	-	His-_14_ tagging
Primer 2	CAACTTCCTAAGCTTCACGCC	HindIII	His-_14_ tagging
Primer 3	GCTGGAGCTCTAGTACG	SacI	His-_14_ tagged subcloning
Primer 4	GCAAATTAAAGCCTTCGA	-	His-_14_ tagged subcloning
Primer 5	AAGCTTAAGCTTGCTATAAGCTAGAATGTATTGAATGTATTC	-	Loop 5 SOE
Primer 6	GGATCCGCGCATTTGCTTAAGCAAGGATACC	HindIII	Loop 5 SOE
Primer 7	GGAAAAAGGCAGGAGCAAACG CTCTCCAG	BamHI	Loop 5 SOE
Primer 8	CGTTTGCTCCTGCCTTTTTCCATCTTTTGGCTG	-	Loop 5 SOE
Primer 9	GGACAAGAAATACCGTACCAATCTACCTAATATGTTCC	-	Loop 5 SOE
Primer 10	TGGTACGGTATTTCTTGTCCT TTCTGACAGCC	-	Loop 5 SOE
Primer 11	CCTTTGTCGACTAGTGTCATGTG	SpeI	*MPD1* HA tagging
Primer 12	GCAGCGAGGTACCGTAATTTTTGC	KpnI	*MPD1* HA tagging
Primer 13	GGATACAAGTCGACGCAAATTTCTC	SalI	*MPD1-HA* subcloning
Primer 14	CAATTTTTGGATGGGAATTCAATTATAC	EcoRI	*MPD1-HA* subcloning

**Table 4:** Antibodies used in this study.

Name	Source	Dilution
Anti-Sec61(N-terminus)	Römisch lab	Western Blot 1: 2.500; IP 1:100
Anti-Sec61(C-terminus)	Römisch lab	Western Blot 1: 2.500; IP 1:100
Anti-Sec63	Schekman lab	Western Blot 1:2.500; IP 1:100
Anti-Rpn12	Römisch lab	Western Blot 1:2.500
Anti-Hrd1	Sommer lab	Western Blot 1:10.000
Anti-Hrd3	Sommer lab	Western Blot 1:10.000
Anti-HA	BioLegend	Western Blot 1:5.000; IP 1:200
Anti-CPY	Römisch lab	IP 1:100
Anti-ppαF	Römisch lab	IP 1:100
Anti-DPAPB	Stevens lab	IP 1:100
Anti- rabbit (HRP)	Rockland	Western Blot 1:10.000

### Growth of *S*. *cerevisiae*

*S*. *cerevisiae* cells were grown at 30°C in YPD or in SC medium with continuous shaking at 220 rpm. Cells on solid medium were also grown at 30°C if not stated otherwise. To test temperature sensitivity, cells were counted and serial dilutions were prepared. A volume of 5 μl of each dilution (containing 10^4^–10 cells) was pipetted onto YPD plates. To test tunicamycin (Tm) (SIGMA) sensitivity, cells were grown on YPD plates supplemented with 0, 0.25 or 0.5 μg/ml Tm. Plates were incubated at indicated temperatures for 3 days.

### Yeast Microsome preparation

The isolation of rough microsomal membranes from *S*. *cerevisiae* was done as in [11] and membranes aliquoted at an *OD*_280_ = 30, snap-frozen in liquid nitrogen, and stored at -80°C. Microsome amounts are referred to as equivalents (eq) in which 1 eq = 1 μl of microsomes at an *OD*_280_ of 50 [29].

To prepare radiolabeled ER vesicles, 7 *OD*_600_ of early log-phase cells were incubated in synthetic minimal media supplemented appropriately and lacking methionine, cysteine, and ammonium sulfate for 30 min at 30°C, 220 rpm. Cells were labelled with 6.5 MBq [^35^S]- methionine/cysteine (Express Labeling, PerkinElmer) mix for 30 min. After labelling, cells were immediately washed twice with Tris-Azide Buffer (20 mM Tris-HCl, pH 7.5, 20 mM sodium azide). Cells were then incubated in100 mM Tris-HCl, pH 9, 10 mM DTT for 10 min at room temperature, sedimented, and resuspended in 300 μl of 2 x JR Lysis Buffer (40 mM Hepes-KOH, pH 7.4, 400 mM sorbitol, 100 mM KOAc, 4 mM EDTA, 1 mM DTT, 1 mM PMSF) [11]. Acid-washed glass beads (1/2 volume) were added and the sample submitted at 2 cycles of 1 min bead-beating (Mini-beadbeater-16, BioSpec) with 2 min of incubation on ice after each cycle. From this point on, all samples were kept at 4°C. Beads were washed 3 times with 300 μl of B88, pH 7.2 (20 mM Hepes-KOH pH 6.8, 250 mM sorbitol, 150 mM KOAc, 5 mM Mg(OAc)_2_). Washes were pooled and sedimented for 2 min at 1,500 x g and the microsome-containing supernatant was transferred to a clean tube. Microsomes were then sedimented at 16,000 x g for 10 min, washed and resuspended in 200 μl B88, pH 7.2. Crude radiolabelled ER vesicles were then aliquoted (50 μl), flash frozen in liquid nitrogen, and stored at -80°C.

### Chemical crosslinking

Microsomes (17 eq) were washed and resuspended in B88 (20 mM Hepes-KOH, 250 mM sorbitol, 150 mM KOAc, 5 mM Mg(OAc)_2_). For SMPH and LC-SPDP crosslinking B88 was used at pH 7.2, for SDAD crosslinking pH was 7.9. The total reaction volume for subsequent detection by immunoblotting was 100 μl with appropriate amount of crosslinker (SMPH or LC-SPDP: 1 mM; SDAD: 1.5 mM). Control reactions were prepared with 5 μl of DMSO, but otherwise treated identically. For up-scaling, proportion of microsomes/total volume was maintained. After crosslinker addition, samples were incubated on ice for 30 min. Then, Quenching Buffer (1M Tris-HCl, pH 8.0; 100 mg/ml L-cys) was added (1/10 of total volume), and the sample incubated on ice for 15 min. Samples were then washed twice (always in the presence of quenching buffer) with appropriate pH B88, membranes sedimented at 16,000 x g for 10 min, and resuspended in appropriate form for subsequent use. For LC-SPDP cleavage, membranes were incubated for 15 min at room temperature in the presence of 100 mM of DTT. For SDAD crosslinking, after the washes the sample was exposed, on ice, to a 15 min UV (365 nm) irradiation with a 3UV Lamp (115V, 60Hz) (ThermoFisher) at a distance of 3,6 cm.

### Extraction of luminal and cytosolic microsome-associated proteins

For extraction of cytosolic membrane-associated proteins, microsomes were resuspended in B88/Urea (20 mM Hepes-KOH, pH 6.8, 250 mM sorbitol, 150 mM KOAc, 5 mM Mg(OAc)_2_, 2.5 M urea), incubated for 20 min on ice, followed by sedimentation and washing of the membranes with B88, pH 6.8. For extraction of ER-luminal proteins, microsomes were resuspended in 100 mM sodium carbonate, pH 11.5, incubated on ice for 20 min, followed by sedimentation (20 min at 346,000xg, 4ºC) of the membranes through a sucrose cushion (200 mM sucrose, 100 mM sodium carbonate, pH 11.5), and resuspension in B88, pH 6.8. For mock extractions, samples were treated in same way, but in absence of either urea or sodium carbonate.

### Immunoblotting

Protein gel electrophoresis was conducted using NuPAGE Novex pre-cast Bis-Tris gels (4–12.5% gels, 1.0 mm) and the XCell SureLock Mini-Cell (both Invitrogen). Proteins were transferred to nitrocellulose membranes (BioRad) and detected with specific antibodies at the appropriate dilutions, and an ECL reagent (Pierce) according to the supplier’s instructions. Signal was acquired either using an Amersham Imager 600 (GE Healthcare) or exposure to ECL films (Adavnsta).

### Purification of Sec61

ER membranes (500 eq) were treated as described in "Chemical Crosslinking", either with DMSO (control), SMPH, or LC-SPDP in a total volume of 1.5 ml. After washing, membranes were resuspended in 150 μl of Quenching Buffer (1 M Tris-HCl, pH 8.0; 100 mg/ml L-cys) and diluted with 1 ml of IP Buffer (15 mM Tris-HCl, pH 7.5, 150 mM NaCl, 1% Triton X-100, 0.1% SDS) for solubilization (30 min at 4°C) followed by 10 min denaturation at 65°C. From this point on, all steps were done at 4°C. Sample was diluted with cold Binding Buffer (50 mM Tris-HCl, 300 mM KCl, 0,5% Triton X-100, 40 mM imidazole) to a final volume of 5 ml and applied to an HisTrap FF crude (1 ml) column integrated into a BioLogic automated purification system (Biorad). After sample loading (0.5 ml/min for 10 ml), the column was washed with Binding Buffer (10 ml; 1 ml/min) and sample eluted along a step gradient of imidazole (100–500 mM, 15 ml per step, 1ml/min. Steps: 100; 200; 400; 500). Fractions (7,5 ml) were collected along the gradient with an automatic fraction collector. DTT (100 mM) was added to each fraction. Each differently treated sample was applied to an independent column. Between purifications, the system was washed with 10 ml H_2_O, 10 ml ethanol 20%, 10 ml H_2_O, 20 ml Binding Buffer. Fractions where Sec61 was eluted (fraction 3–10–50 ml total) were pooled, proteins precipitated with 10% TCA on ice for 2h and washed with ice-cold acetone. Each pellet was resuspended in 2 x Laemmli Buffer, and resolved for 5 cm on 4–12,5% NuPAGE gel. The gel was then stained by Coomassie Colloidal Staining (0.08% Coomassie Brilliant Blue G250 (CBB G250), 10% citric acid, 8% ammonium sulfate, 20% methanol) overnight and destained with water as described in the EMBL online Proteomics Core Facility Protocols. The gels where then sealed in individual plastic bags with a few milliliters of water and shipped to the Mass Spectrometry Facility.

### Mass spectrometry

#### Sample preparation

The whole lane of each samples was cut out into small cubes and subjected to in-gel digestion with trypsin [30]. After overnight digestion, peptides were extracted from the gel pieces by sonication for 15 minutes, tubes were centrifuged, the supernatant removed and placed in a clean tube. Followed by a second extraction round with a solution of 50:50 water: acetonitrile, 1% formic acid (2 x the volume of the gel pieces) and the samples were sonicated for 15 minutes, centrifuged and the supernatant pooled with the first extract. The pooled supernatants were then subjected to speed vacuum centrifugation. Samples were reconstituted in 96:4 water: acetonitrile, 0.1% formic acid and further processed using an OASIS HLB μElution Plate (Waters) according the manufacturer’s instructions.

#### LC-MS/MS

Peptides were separated using the nanoAcquity Ultra Performance Liquid Chromatography (UPLC) system (Waters) using a trapping (nanoAcquity Symmetry C18, 5 μm, 180 μm x 20 mm) as well as an analytical column (nanoAcquity BEH C18, 1.7 μm, 75 μm x 200 mm). The outlet of the analytical column was coupled to a Linear Trap Quadrupole (LTQ) Orbitrap Velos Pro (Thermo Fisher Scientific) using the Proxeon nanospray source. Solvent A consisted of water, 0.1% formic acid and solvent B consisted of acetonitrile, 0.1% formic acid. Sample was loaded with a constant flow of solvent A at 5 μl/min onto the trapping column. Peptides were eluted over the analytical column with a constant flow of 0.3 μl/min During elution the percentage of solvent B increased linearly from 3% to 7% in 10 min., then increased to 25% in 110 min and to 40% for the final 10 min a cleaning step was applied for 5 min with 85% B followed by 3% B 20 min. The peptides were introduced into the mass spectrometer via a Pico-Tip Emitter 360 μm OD x 20 μm ID; 10 μm tip (New Objective), a spray voltage of 2.2 kV was applied. Capillary temperature was 300°C. Full scan MS spectra were acquired with a resolution of 30000. The filling time was set at a maximum of 500 ms with a maximum ion target of 1.0 x 10^6^. The fifteen most intense ions from the full scan MS (MS1) were sequentially selected for sequencing in the LTQ. Normalized collision energy of 40% was used, and the fragmentation was performed after accumulation of 3.0 x10^4^ ions or after a maximum filling time of 100 ms for each precursor ion (whichever occurred first). Only multiply charged (2^+^, 3^+^, 4^+^) precursor ions were selected for MS/MS. The dynamic exclusion list was restricted to 500 entries with maximum retention period of 30 s and a relative mass window of 10 ppm. In order to improve the mass accuracy, a lock mass correction using the ion (m/z 445.12003) was applied.

#### Data analysis

The raw mass spectrometry data was processed with MaxQuant (v1.5.2.8) [31] and searched against an Uniprot Saccharomyces cerevisiae proteome database. The search parameters were as follows: Carbamidomethyl (C) (fixed), Acetyl (N-term) and Oxidation (M) (variable) were used as modifications. For the full scan MS spectra (MS1) the mass error tolerance was set to 20 ppm, and for the MS/MS spectra (MS2) to 0.5 Da. Trypsin was selected as protease with a maximum of two missed cleavages. For protein identification a minimum of one unique peptide with a peptide length of at least seven amino acids and a false discovery rate below 0.01 were required on the peptide and protein level. The match between runs function was enabled, a time window of one minute was set. Label free quantification was selected using iBAQ (calculated as the sum of the intensities of the identified peptides and divided by the number of observable peptides of a protein) [32] with the log fit function enabled. We also used the xQuest/xProphet pipeline [33] to identify crosslinked peptides in our samples. For this, we used the basic protocol and conditions used in [33], correcting the meaningful parameters to fit our crosslinker (e.g monoisotopic shift, only light chain, reactive groups, etc.). Databases of no more than 30 proteins were fed into the pipeline.

This software identifies and statistically validates crosslinked peptides from XL-MS experiments. The software was not optimized for our crosslinking setup, but in fact none of the available software was. There are two main reason for the inadequacy of most software: 1) The complexity of our sample was high: A total of 1900 different proteins were identified by mass spectrometry in the 12 samples analyzed. 2) The crosslinker used: Most software is optimized for the analysis of samples treated with homobifunctional crosslinkers like DSS. So although a reactivity to both lysine (K) and cysteine (C) could be set, the software was not able to exclude homocrosslinks (K-K or C-C); these needed to be excluded manually. A general schematic for our use of xQuest/xProphet is shown in S2 Fig. During our analyses, a specific interaction prediction appeared multiple times, the interaction between Sec61 and Mpd1. The software reported a specific crosslink between Mpd1 C59 and Sec61 K209 (K243 in the 14His-sec61S353C mutant) (S3A Fig). This particular analysis was done with a database comprising Sec61, Pbr1 (Yns1), Sec63, YNL021W (Yn8b), Asi3, She2, Psg1 (Ykh7) and Mpd1. In this analysis, several potential Sec63 crosslinking sites were also detected, as well as potential interactions with the other tested hits (S3B Fig).

#### Statistical analysis

The raw output data of MaxQuant (proteinGroups.txt file) was processed using the R programming language (ISBN 3-900051-07-0). As a quality filter we allowed only proteins that were quantified with at least 2 unique peptides. Potential batch-effects were removed from the log2 of the iBAQ values using the limma package [34]. Furthermore, batchcleaned data were normalized with the vsn package (variance stabilization) [35]. Missing values were imputed using the MSNbase package [36]. For conditions with at least 2 out of 3 identifications, the “knn” method was used. For less identifications, the “MinDet” method was applied. Finally, limma was used again to identify differentially expressed proteins. A protein was called a hit with a false discovery rate (fdr) smaller 5% and a fold change of at least 3 and a candidate with an fdr smaller 20% and a fold change of at least 3.

### Mutant construction

#### 14His-Tagged constructs

For His_14_-tagging of *SEC61* and *sec61S353C*, both genes were amplified from pBW11 and pRS315- *sec61S353C*, respectively, using Primer 1 and Primer 2. The resulting PCR products were cloned into pRS426pGAL1 [25] using the SfoI and HindIII restriction sites. Correct cloning was confirmed by sequencing. The pGal-His_14_-*SEC61*-CYC and pGal-His_14_-*Sec61S353C*-CYC cassettes were then amplified using Primer 3 and Primer 4. The resulting PCR products were cloned into pRS315 (CEN, *LEU2*). Transformants in the JDY638 (pGAL-*SEC61-URA3*) *S*. *cerevisiae* background were first selected on SC -URA medium containing 2% (w/v) galactose and 0.2% (w/v) glucose lacking leucine. The pGAL-*SEC61* plasmid was selected against on SC 5-FOA plates containing 2% (w/v) galactose and 0.2% (w/v) glucose without leucine. Constructs were confirmed by sequencing.

#### *SEC61* Loop 5 deletion mutants

Mutants *sec61del1*, *sec61del2*, and *sec61del1/2* were generated by PCR-driven overlap extension (SOE PCR) [37,38] followed by transformation into KRY461 of the respective constructs. For the initial SOE-PCR reactions, *SEC61* was amplified from pBW11 (Table 2). Deletion 1 and deletion 2 were made separately. Deletion 1/2 was made using deletion 1 construct as template and same primers as used for the generation of deletion 2. For SOE-PCR, the regions upstream and the downstream of the deletion sites were amplified using a mutagenic primer and a gene flanking primer (Table 3). Each mutagenic primer immediately flanks the deletion site and both upstream and downstream deletion-flanking primer have a stretch of complementarity with each other. For the extension of the final PCR product, the gene-flanking primer-pair was used and both upstream and downstream fragments were used as template (working as a single-template unit). The resulting PCR products were cloned into pRS315 (CEN, *LEU2*) [23]. Transformants into JDY638 (*pGAL-SEC61-URA3*) were first selected on SC -URA medium containing 2% (w/v) galactose and 0.2% (w/v) glucose without leucine. The *pGal-SEC61* plasmid shuffle was done on SC 5’-FOA plates lacking leucine. All constructs were confirmed by sequencing.

#### *MPD1* HA-Tagging

Tagging of genomic *MPD1* was done as described in [28]. Briefly, the HA cassette was amplified from pYM24 (supplied by Michael Knop) using Primer11 and Primer12. The plasmid contains the HA-cassette as well as the hphNT1 for selection. Targeting was done by homology of the designed primers with the appropriate regions of the gene of interest. This PCR product was then used to transform KRY461, and transformants were selected on YPD plates containing hygromycin (300 μg/ml). *MPD1-HA* was amplified from the genomic DNA using Primer13 and Primer14 and cloned into pRS426 (2μ, *URA3*). This plasmid was then used to transform the hinge mutant strains.

### Cell labelling and immunoprecipitation

Aliquots of 1.5 OD_600_ early log phase cells were incubated in synthetic media lacking methionine, cysteine, and ammonium sulfate for 15 or 30 min (depending on the protein to be labelled) at the appropriate temperature and shaking at 220 rpm. Cells were labeled with [^35^S]-met/cys (Express Labeling, PerkinElmer) (1.5 MBq per sample) mix for 5 min (CPY*, pΔgpαF) or 15 min (DPAPB, KWW, KHN). For pulse experiments, after labeling cells were immediately killed with Tris-Azide Buffer (20 mM Tris-HCl, pH 7.5, 20 mM sodium azide). For pulse-chase experiments, zero time points were treated as above, and to remaining samples Chase Mix (0.03% cys, 0.04% met, 10 mM ammonium sulfate) was added, and samples were incubated with shaking at the appropriate temperature for the indicated times. At each time point, Tris-Azide Buffer was added. Cells were harvested and incubated in 100 mM Tris-HCl, pH 9.4, for 10 min at room temperature. Subsequently, samples were lysed with glass beads in Lysis Buffer (20 mM Tris-HCl, pH 7.5, 2% (w/v) SDS, 1 mM DTT, 1 mM PMSF) and denatured for 5 min at 95°C (soluble proteins) or 10 min at 65°C (transmembrane proteins). Afterwards, glass beads were washed 3 times and the combined washes used for immunoprecipitation after preclearing with 60 μl 20% Protein A-Sepharose beads (GE Healthcare) in IP-buffer (15 mM Tris-HCl, pH 7.5, 150mM NaCl, 1% Triton X-100, 0,1% SDS) [11]. Precipitations were done with 60 μl 20% Protein A-Sepharose beads (GE Healthcare) and appropriate amount of antibody, either at room temperature for 2 h or at 4°C for 4 h or over night. Protein A-Sepharose beads were washed as in Baker et al. (1988), proteins eluted with 2x Laemmli Buffer and denatured at 95°C for 5 min (soluble) or 65°C for 10 min (transmembrane). Proteins were resolved on 4–12,5% NuPAGE gels. Dried gels were exposed to Phosphorimager plates, and the signal acquired with a Typhoon PhosphoImager (GE Healthcare).

### Detection of Sec61 interactors in radiolabeled membranes

Crude radiolabeled ER vesicles (10 μl) were crosslinked as described in "Chemical Crosslinking" and submitted to two consecutive immunoprecipitations. Hinge mutants are derived from a *SEC61* background. Microsomes from the *sec61S353C* strain were included, because Sec61-Mpd1 interaction was first detected in this strain. Crosslinker selection: The Sec61-Mpd1 crosslinked peptide was first identified by SMPH crosslinking to Sec61S353C. SMPH and LC-SPDP have one cysteine- and one NH_2_-reactive group. Only LC-SPDP is cleavable, so in the double immunoprecipitation experiment, SMPH is the negative control for LC-SPDP, because there should be no release of Mpd1 from Sec61 after the first precipitation. SDAD is also cleavable, but with one NH_2_-reactive and one photoactivatable reactive group. It was used to efficiently crosslink Mpd1 to Sec61 regardless of the cysteine in loop 7. For the first precipitation, the membranes were solubilized in Lysis Buffer (20 mM Tris, pH 7.5, 2% SDS, 1 mM PMSF) and denatured at 65°C for 10 min. Proteins were then diluted in Washing Buffer (15 mM Tris-HCl, pH 7.5, 150 mM NaCl, 1% Triton X-100, 2 mM NaN3, 1 mM PMSF). After pre-clearing (as previously), 60 μl of 20% Protein A-Sepharose beads (GE Healthcare) and a saturating amount of Sec61 antibody was added (S4 Fig). Samples were then incubated with rotation overnight at 4°C, and Protein A-Sepharose pellets washed as above. For elution we used 20 μl of 20 mM Tris-HCl, pH 7.5, 5% SDS, 50 mM DTT for 15 min room temperature and denaturation for 10 min 65°C. Eluted proteins were then diluted in Washing Buffer and the Mpd1-HA precipitated using anti-HA polyclonal antibody (BioLegend). Precipitation was done for 2h at room temperature followed by elution done 2 x Laemmli Buffer, 200 mM DTT. Proteins were denatured again as before, resolved on 4–12.5% NuPAGE gels exposed to Phosphorimager plates, and the signal acquired with a Typhoon PhosphoImager (GE Healthcare).

## Results and discussion

### Sec61 crosslinking, purification, and mass spectrometry

**Fig 1:**
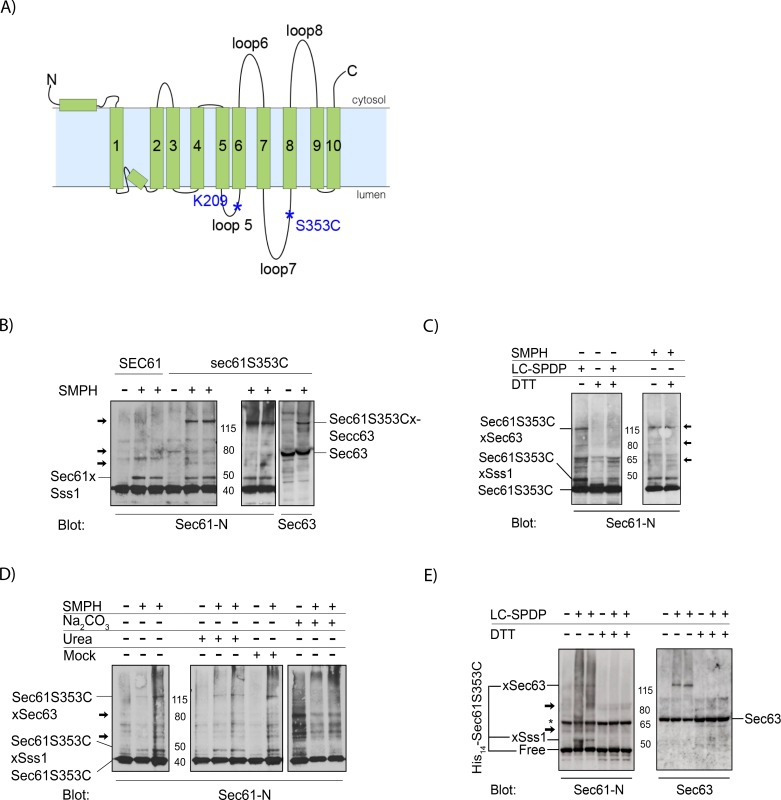
Optimization of crosslinking to Sec61S353C. **A:** Topological model of Sec61. **B:** Comparison of crosslinking patterns to Sec61 versus Sec61S353C with cysteine- and NH2-reactive SMPH. 17 eq microsomes per lane were crosslinked with 1 mM SMPH on ice and proteins resolved by SDS-PAGE. Sec61 was detected with an antibody against its N-terminus. Note that both Sec61 and Sec61S353C crosslink to Sss1. Additional crosslinked bands occurring in Sec61S353C samples are indicated by arrows in Sec61 panel. The largest product consists of Sec61S353C crosslinked to Sec63 (right panel). **C:** Sec61S353C crosslinking with SMPH (non-cleavable) or LC-SPDP (cleavable). Crosslinking was done as above and samples were resolved on SDS-PAGE without or with 200 mM DTT in the sample buffer as indicated. **D:** Crosslinking to Sec61S353C after microsome extraction. Microsomes (17 eq/lane) were extracted as indicated or mock-treated, crosslinked as above, and Sec61S353C and crosslinking products detected with an antibody against the Sec61 N-terminus. Note that crosslinks to Sss1 and Sec63 are sensitive to carbonate-extraction. **E:** Crosslinking of His14-Sec61S353C microsomes with LC-SPDP. Crosslinking was done as above. Note that the N-terminal His14-tag did not affect crosslinking to Sec63 or Sss1 indicating no gross conformational alterations in the Sec61 complex. The asterisk indicates a non-specific band occuring independently of crosslinking in the Sec61 blot.

To identify new lumenal interaction partners of Sec61 we used a functional *sec61* mutant with a unique cysteine in its large lumenal loop 7 (Fig 1A) [13]. The mutant protein is stable and expressed at wildtype levels, and cells expressing *sec61S353C* are fully import competent and do not display any growth defects [13]. Using heterobifunctional non-cleavable (SMPH) or cleavable (LC-SPDP) crosslinkers with a cysteine-reactive and an amino-reactive group to crosslink yeast microsomes, as described in Materials & Methods, we found additional bands in the crosslinking patterns to Sec61S353C compared to wildtype Sec61—suggesting bound lumenal interactors (Fig 1B and 1C, arrows). Amongst those was Sec63, a well characterized J-domain protein that contributes to both co- and posttranslational import into the ER and to ERAD (Fig 1B and 1C, arrows) [4,39]. While pretreatment of microsomes with urea had no effect on the Sec61S353C-associated proteins (Fig 1D, lanes 4–6), extraction of microsomes with sodium carbonate resulted in reduced crosslinking to Sss1 which is known to be partially carbonate-extractable [40] and to Sec63 (Fig 1D, lanes 10–12). Our data suggest an interaction between the Sec63 lumenal J-domain or N-terminus with Sec61 loop7.

**Fig 2:**
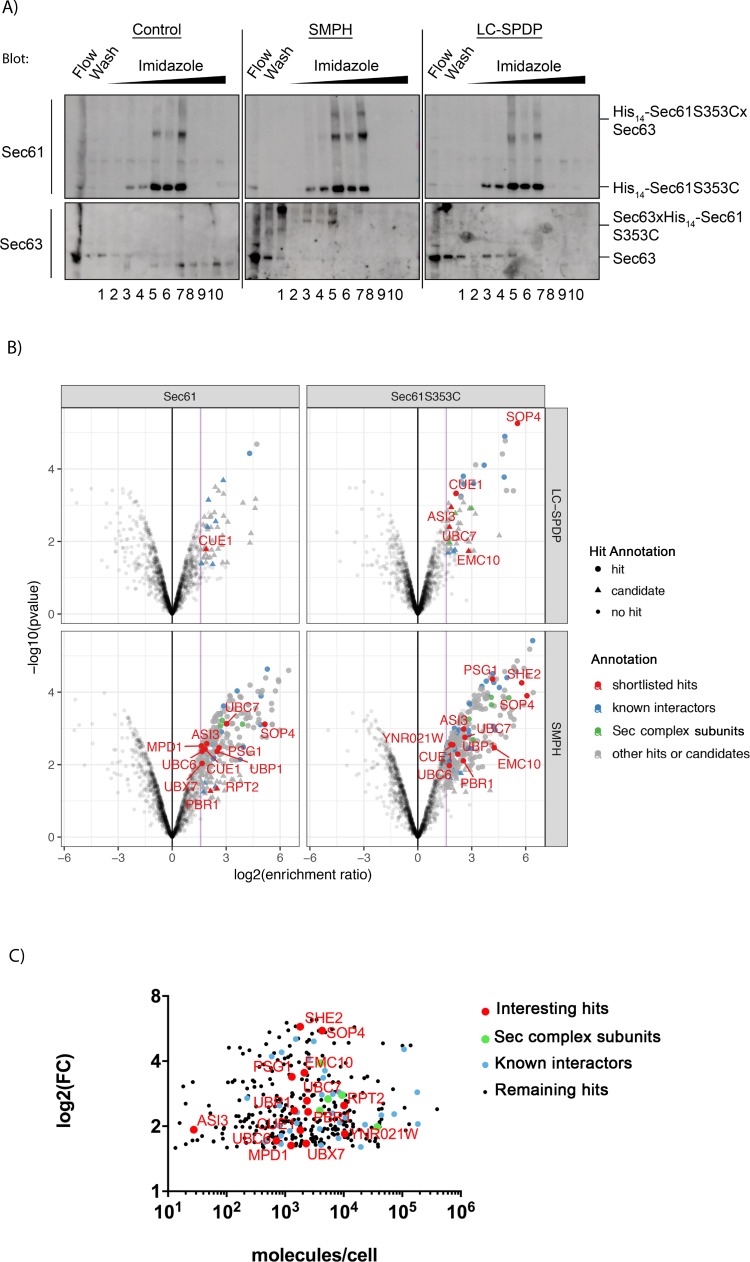
Purification and proteomics of Sec61-crosslinked proteins. **A:** 500 eq microsomes treated with DMSO (control), SMPH (non-cleavable), or LC-SPDP (cleavable) were used for purification. Crosslinking was quenched and membranes solubilized in IP Buffer. After denaturation (10 min, 65°C) proteins were diluted with cold Binding Buffer and applied to a HisTrap FF crude 1 ml column. Proteins were eluted with an imidazole gradient (100–500 mM). Sec61 and Sec63 were detected in each fraction without (SMPH) or after cleavage (LC-SPDP) and gel electrophoresis by immunoblotting with specific antibodies. **B:** Volcano plots based on statistically determined protein enrichment in the crosslinked His14-Sec61 and His14-Sec61S353C samples compared to non-crosslinked samples. Horizontal axis represents log2-fold change (log2FC) relecting level of enrichment. Vertical axis plots -log10(p value) of enrichment reflecting significance. Both hits and candidates have fold change of at least 3, indicated by purple line. Hits (colored dots) have a false discovery rate (FDR)< 5%, candidates (colored triangeles) an FDR < 20%. Sec complex subunits in green, known Sec61 interactors and translation machinery in blue, shortlisted hits in red with points labeled on graph. Non-significant hits below reference line and non-interesting hits above in grey. **C:** Enrichment of Sec61 interactors as function of respective cellular abundance (as in [48]). Color coding as above. Note absence of correlation between abundance and Sec61 interaction.

For enrichment of Sec61-crosslinked proteins we tagged the N-termini of Sec61 and Sec61S353C with 14-His which had no effects on growth, expression levels, or tunicamycin-sensitivity and UPR induction (not shown), indicating no perturbance of ER proteostasis. Crosslinking patterns were not affected by the tagging (Fig 1E). Sec61- and Sec61S353C-crosslinked proteins were purified from 500 eq lysed microsomes on a nickel column and eluted with imidazole (Fig 2A). Fractions 3–10 of the eluates were pooled and proteins analyzed by mass spectrometry. Proteins were accepted as interactors if there was at least a 3-fold enrichment compared to the uncrosslinked sample (Fig 2B). In total, we identified 353 proteins that were copurifying with Sec61in the crosslinked samples (S1 Table). While the enrichment pattern was sample- and crosslinker-dependent (S1 Table), the absolute abundance of proteins in the ER did not affect interaction with Sec61 (Fig 2C) suggesting that the interactions we detected were specific. We detected all subunits of the Sec complex in the ER membrane, SRP receptor, Snd3, and several subunits of oligosaccharyl transferase (S1 Table). In the same significance range we found a number of new interaction partners of Sec61 that were ERAD relevant: Asi3, Ubc6, Ubc7, Cue1, Ubx7, Ubp1, Rpt2, ER-membrane complex (EMC) subunits, and Mpd1, suggesting close physical contact of the Sec61 channel with the ERAD machinery [41, 9, 42, 17, 43–45].

### Mpd1-Sec61 interaction

**Fig 3:**
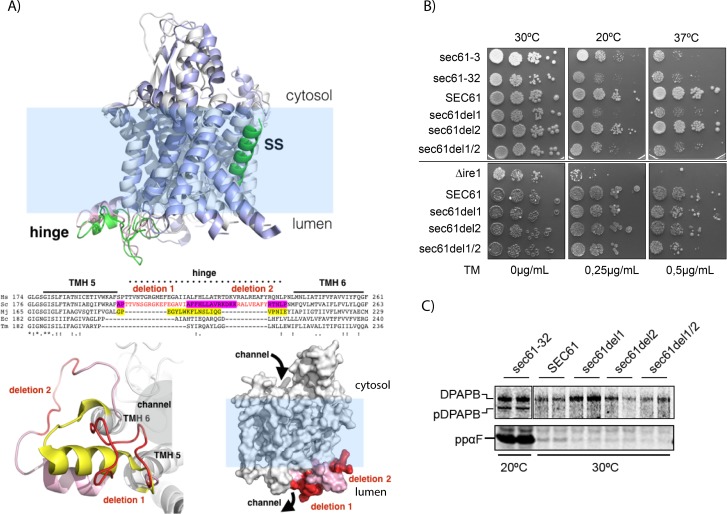
Design and characterization of *sec61* loop5 hinge mutants encompassing the binding site for Mpd1. **A:** Top: Structure of the Sec61 channel in closed (grey helices, pink hinge) versus open state (blue helices, green hinge, green signal sequence (SS) inserted in lateral gate; PDB 3J7Q, 3J7R) [47]. Note conformational change in hinge (pink vs. green) during channel opening. Middle: Alignment of loop5 hinge sequences of eukaryotes (*Homo sapiens*, Hs; *Saccharomyces cerevisiae*, Sc), prokaryotes (*Escherichia coli*, Ec; *Thermotoga maritima*, Tm), and archaea (*Methanococcus jannaschii*, Mj). Protein sequences from Uniprot. Regions coded by deletions in *sec61* hinge mutants in red. Sequence forming the archaeal hinge region in yellow, sequence corresponding to vestigial (post-deletion) eukaryotic counterpart in magenta. Bottom left: hinge (eukaryotic, PDB 3J7Q) from the ER lumen showing the protein channel lined by TMHs 5 along with 6, and the intervening hinge (pink) with deletions 1 and 2 in red. The deletions result in a shorter hinge akin to the archaeal structure shown in yellow (PDB 1RHZ; also see middle). Bottom right: space filling model of Sec61 channel (PDB 3J7Q) in ER membrane indicating positions of deletions 1 and 2. Note that region deleted in *sec61del1* is accessible for lumenal proteins in contrast to *sec61del2* which faces the membrane. **B:** Growth of *SEC61* and *sec61* hinge mutants at different temperatures (as indicated, top), or in the presence of tunicamycin (as indicated; at 30°C; bottom). Cells (104–10) were grown on YPD plates for 3 days. The *sec61-3*, *sec61-32*, and *Δire1* strains are shown as controls. **C:** Analysis of ER import in *sec61* hinge mutants. Early log phase cells were pulse-labelled with [^35^S]-met/cys, lysed, and immunoprecipitations of DPAPB (upper, cotranslational import) or prepro alpha factor (ppαF, posttranslational import, lower) performed. Proteins were detected by phosphorimaging. Starving and labelling were done at 30°C except for *sec61-32* which was incubated at 20°C. Labelling was 15 min for DPAPB, 5 min for ppαF.

**Fig 4:**
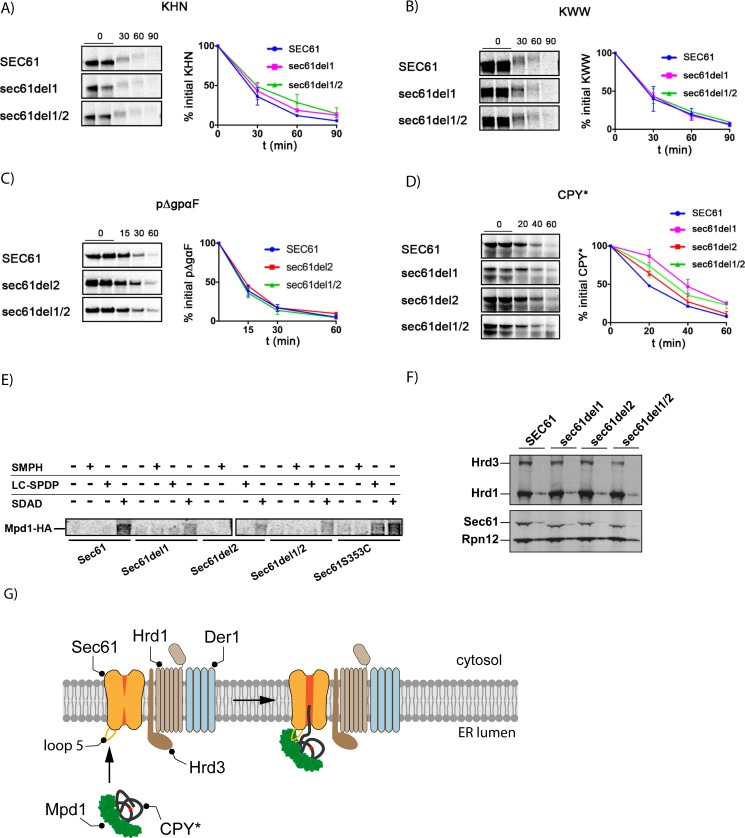
Mutation of the loop5 hinge in Sec61 specifically affects CPY* ERAD and interaction with Mpd1. **A—D:** We investigated ERAD of KHN, KWW, pΔgpαF, and CPY* in our *sec61* hinge mutants. Wildtype and mutant strains were labelled with [^35^S]-met/cys for 5 min (pΔgpαF, CPY*) or 15 min (KHN, KWW) followed by chase for indicated times. At each time point 1.5 OD_600_ of cells were lysed and proteins immunoprecipitated with specific antibodies (pΔgpaF, CPY*), or anti-HA (KHN, KWW). After SDS-PAGE proteins were detected by phosphorimaging, quantified by ImageQuant (GE Healthcare), and averaged values of at least 3 replicas plotted. **E:** Interaction of Sec61 with Mpd1 was determined by crosslinking in [^35^S]-met/cys-labelled microsomes with SMPH (cys- and NH_2_-reactive, non-cleavable), LC-SPDP (cys- and NH_2_-reactive, cleavable), or SDAD (NH_2_- and photo-reactive, cleavable). For explanation of crosslinker selection, see Materials & Methods. Sec61 and crosslinked proteins were precipitated with anti-Sec61 N-terminal antibodies, followed by reduction which cleaves LC-SPDP and SDAD. Subsequently, Mpd1-HA released from Sec61 by cleavage was precipitated from the supernatant with anti-HA antibodies. After gel electrophoresis, Mpd1-HA was detected by phosphorimaging. Equal amounts of cells were used for preparation of each microsome batch. Protein levels of both Sec61 and Mpd1-HA were similar in all strains. Saturating amounts of antibodies were used in each precipitation. **F:** Steady-state levels of Sec61, Hrd1, and Hrd3 were determined by immunoblotting in extracts of wildtype and *sec61* hinge mutant cells. Two amounts of each sample (1 and 1/3) were loaded side by side with Rpn12 as loading control. We used specific antibodies for each protein. **G:** Model for initiation of CPY* ERAD mediated by Mpd1 interaction with lumenal hinge of Sec61.

We then decided to investigate the interaction of Se61 with Mpd1, a PDI homolog and known ERAD factor of the well-characterized ERAD substrate CPY* [46,45]. Our xQuest/xProphet analysis of crosslinked peptides suggested a direct interaction of Mpd1 C59—the first cysteine in its single redox-active CXXC motif—with K209 in lumenal loop5 of Sec61 which constitutes the hinge region around which the N-terminal half of Sec61 swings during channel opening (Fig 3A, upper) [33,46,47]. Comparison of Sec61 loop5 with loop5 in SecY of bacteria and archaea revealed a substantial extension of loop5 in eukaryotes including the crosslinking site to Mpd1 (Fig 3A, middle and lower). We hypothesized that the eukaryotic extensions in loop5 might serve as docking sites for ERAD factors to facilitate opening of the Sec61 channel from the lumen for export of ERAD substrates. To test this hypothesis we deleted sections of the Sec61 hinge including the Mpd1 contact site to create a smaller vestigial hinge within Sec61, similar to the SecY counterpart (Fig 3A, middle and lower), and investigated the effects on protein transport into the ER and ERAD. While deletion1 caused temperature- and cold-sensitivity alone and in combination with deletion2 (Fig 3B), steady-state expression levels of all hinge mutants were like wildtype (Fig 4F), and there was no effect on co- or posttranslational protein import into the ER (Fig 3C).

As only the double mutant *sec61del1/2* showed a moderate tunicamycin-sensitivity (Fig 3B) and slightly induced UPR (S2 Fig), ER-proteostasis was not dramatically compromised in the mutants excluding gross ERAD defects. This was confirmed by normal ERAD kinetics for the KHN, KWW, and pΔgpαF substrates in the mutants (Fig 4A, 4B and 4C) [11,27]. CPY* degradation, however, was compromised in *sec61del1* which lacks the contact site for Mpd1 (Fig 4D, magenta). In contrast, *sec61del2* barely affected CPY* degradation (Fig 4D, red). The *sec61del1/2* mutant had an intermediate phenotype (Fig 4D, green) which may suggest that it was not just the absence of specific amino acids deleted in *sec61del1*, but also the distortion of the hinge by the deletion that caused the CPY* ERAD defect (Fig 3A, lower). In *sec61del1/2* this distortion is partially compensated (Fig 3A, lower). KHN and KWW both contain disulfide bonds like CPY*, whereas pΔgpαF does not contain any cysteines [27,26]. As neither ERAD of soluble KHN nor ERAD of its membrane-anchored counterpart KWW was affected in the *sec61* hinge deletion mutants (Fig 4A and 4B) Mpd1 interaction with Sec61 does not appear to be critical for ERAD of all disulfide-containing proteins, but specifically for the degradation of CPY*.

To directly confirm that the Mpd1 interaction with Sec61 was compromised in the *sec61* hinge mutants, we prepared radiolabelled microsomes from wildtype, *sec61S353C*, and *sec61* hinge mutant strains expressing HA-tagged Mpd1 and performed sequential immunoprecipitations with Sec61 and HA-antibodies. In all hinge mutants less Mpd1 was associated with Sec61 compared to wildtype or Sec61S353C (Fig 4E), but it was not possible to correlate the amount of Mpd1 bound to Sec61 with the degree of the CPY* ERAD defect (compare Fig 4D and 4E). The more dramatic effect of the *sec61del2* mutant on Mpd1 crosslinking than on ERAD may be due to the fact that crosslinking is critically dependent on steric proximity of few amino acids whereas protein-protein interaction is usually via a larger surface area. We were, however, unable to coprecipitate sufficient amounts of Mpd1 with Sec61 under native conditions which is why we resorted to crosslinking. To exclude that the *sec61* hinge mutants reduced biogenesis of the ER ubiquitin ligase Hrd1 and its cofactor Hrd3 we performed quantitative immunoblots for both proteins and found that they were expressed equally in wildtype and *sec61* hinge mutant cells (Fig 4F).

The doubling of the _t1/2_ of CPY* that we observe in *sec61del1* (Fig 4D, magenta) is comparable to the effect of deletion of *MPD1* on CPY* degradation [45] suggesting that Mpd1 primarily promotes CPY* ERAD by its interaction with the Sec61 hinge. We have shown previously that while the oxidoreductase function of protein disulfide isomerase (Pdi1) is critical for ERAD of disulfide-bonded CPY*, Pdi1's chaperone function—which is decisive for ERAD targeting of other substrates—is not [26,45]. Our current data indicate that for CPY* this targeting role may be fulfilled by the PDI homolog Mpd1 (Fig 4G). Mpd1 consists of two thioredoxin modules, a and b [46]. The redox-inactive Mpd1 b module contains an extension similar to, but substantially longer than an extension in the redox-inactive b' module of Pdi1 which serves to bind substrate proteins when it acts as an ERAD targeting chaperone [46,26]. The Mpd1 b domain may therefore be responsible for substrate binding whereas the a domain—containing C59 that we crosslinked to Sec61 loop5—interacts with the Sec61 channel.

## Conclusion

Collectively, our data suggest that interaction of the CPY* ERAD factor Mpd1 with the Sec61 hinge region in loop5 contributes to export and degradation of this substrate. Our results are consistent with the view that Sec61 forms part of an export complex in the ER membrane for misfolded protein transport to the cytosol (Fig 4G). The extended hinge in Sec61 compared to SecY (Fig 3A) may serve to activate and open the channel from the lumen for intercalation and subsequent transport of CPY* to the cytosol (Fig 4G).

## Supporting information

S1 Table Mass spectrometry statistical analysis (determination of hits and candidates). (XLSX)

S1 File (DOC)

S1 Fig *HAC*1 mRNA Splicing Assay to evaluate UPR induction. Wildtype and Sec61 hinge mutants were either treated with tunicamycin (2 μg/ml) (TM) or DMSO (control), followed by total RNA isolation, and cDNA production from isolated RNA. A quantitative PCR was done from equal amounts of cDNA. Agarose gel showing the resultant PCR products. Upper slice shows *HAC1* PCR product. Upper bands (720 bp) represent the unspliced (uninduced) *HAC1* mRNA, while lower bands (470 bp) represent the spliced (induced) *HAC1* mRNA. Bottom slice show the actin PCR product. The Δ*ire1* mutant was used as negative control.
(PDF)

S2 Fig xQuest/xProphet pipeline analysis scheme. Workflow of the xQuest/xProphet software pipeline for the identification and statistical validation of cross-linked peptides from XL-MS experiments. The first step includes the conversion of raw MS data to the mzXML format and the preparation of the folder structure for the xQuest search. The second step includes the xQuest search and the identification of cross-linked peptides. The third step describes the statistical validation of the xQuest search results by xProphet, and the fourth step illustrates the web server–based data and result visualization. UI, user interface.
(PDF)

S3 Fig xQuest/xProphet Sec61xMpd1 crosslinking report. Example of the returned results after xQuest/xProphet analysis. A) Detected Sec61xMpd1 crosslinked site. B) Resume of the detected crosslinked sites detected by the software in a given analysis. A mapping of the detected crosslinked positions onto Sec61 can also be seen.
(PDF)

S4 Fig Immunoprecipitation of Sec61, Mpd1-HA, and Sec63 from radiolabelled crude microsomes. Samples were immunoprecipitated using saturating amounts of anti-Sec61 N-terminus, anti-HA, or anti-Sec63 antibodies. Conditions used for immunoprecipitation were the same as for the first immunoprecipitation done for Mpd1xSec61 interaction determination (Fig 4E) as well as in the same backgrounds. Samples were resolved by SDS-Page and signal acquired by phosphorimaging.
(PDF)

## References

[1] Ghaemmaghami S, Huh W-K, Bower K, Howson RW, Belle A, Dephoure N, et al Global analysis of protein expression in yeast. Nature. 2003;425(6959):737–41. 10.1038/nature02046 14562106

[2] Johnson AE, Waes MAV. The Translocon: A Dynamic Gateway at the ER Membrane. Annual Review of Cell and Developmental Biology. 1999;15(1):799–842. 10.1146/annurev.cellbio.15.1.799 10611978

[3] Kalies K-U, Görlich D, Rapoport TA. Binding of ribosomes to the rough endoplasmic reticulum mediated by the Sec61p-complex. The Journal of Cell Biology. 1994 1;126(4):925–34. 8051212 10.1083/jcb.126.4.925 PMC2120124

[4] Brodsky JL, Goeckeler J, Schekman R. BiP and Sec63p are required for both co- and posttranslational protein translocation into the yeast endoplasmic reticulum. Proceedings of the National Academy of Sciences. 1995 10;92(21):9643–6. 10.1073/pnas.92.21.9643 PMC40858 7568189

[5] Jadhav B, McKenna M, Johnson N, High S, Sinning I, Pool MR. Mammalian SRP receptor switches the Sec61 translocase from Sec62 to SRP-dependent translocation. Nature Communications. 2015;6(1). 10.1038/ncomms10133 PMC4686813 26634806

[6] Scheper W, Thaminy S, Kais S, Stagljar I, Römisch K. Coordination ofN-Glycosylation and Protein Translocation across the Endoplasmic Reticulum Membrane by Sss1 Protein. Journal of Biological Chemistry. 2003;278(39):37998–8003. 10.1074/jbc.M300176200 12860997

[7] Kalies K-U, Rapoport TA, Hartmann E. The β Subunit of the Sec61 Complex Facilitates Cotranslational Protein Transport and Interacts with the Signal Peptidase during Translocation. The Journal of Cell Biology. 1998;141(4):887–94. 9585408 10.1083/jcb.141.4.887 PMC2132780

[8] Pilla E, Schneider K, Bertolotti A. Coping with protein quality control failure. Ann Rev Cell Dev Biol. 2017;33:439–465. 28992440 10.1146/annurev-cellbio-111315-125334

[9] Römisch K. Endoplasmic Reticulum–Associated Degradation. Annual Review of Cell and Developmental Biology. 2005;21(1):435–56. 10.1146/annurev.cellbio.21.012704.133250 16212502

[10] Römisch K. A Case for Sec61 Channel Involvement in ERAD. Trends in Biochemical Sciences. 2017;42(3):171–9. 10.1016/j.tibs.2016.10.005 27932072

[11] Pilon M, Schekman R, Romisch K. Sec61p mediates export of a misfolded secretory protein from the endoplasmic reticulum to the cytosol for degradation. The EMBO Journal. 1997 1;16(15):4540–8. 10.1093/emboj/16.15.4540 9303298 PMC1170080

[12] Tretter T, Pereira FP, Ulucan O, Helms V, Allan S, Kalies K-U, et al ERAD and protein import defects in a sec61 mutant lacking ER-lumenal loop 7. BMC Cell Biology. 2013;14(1):56. 24314051 10.1186/1471-2121-14-56 PMC3897919

[13] Kaiser M-L, Römisch K. Proteasome 19S RP Binding to the Sec61 Channel Plays a Key Role in ERAD. Plos One. 2015 6;10(2). 10.1371/journal.pone.0117260 PMC4319758 25658429

[14] Braunstein I, Zach L, Allan S, Kalies KU, Stanhil A. Proteasomal degradation of preemptive quality contol (pQC) substrates is mediated by an AIRAPL-p97 complex. Mol Biol Cell 2015; 26:3719–27. 10.1091/mbc.E15-02-0085 26337389 PMC4626058

[15] Mehnert M, Sommer T, Jarosch E. Der1 promotes movement of misfolded proteins through the endoplasmic reticulum membrane. Nature Cell Biology. 2013 1;16(1):77–86. 10.1038/ncb2882 24292014

[16] Neal S, Jaeger PA, Duttke SH, Benner CK, Glass C, Ideker T, et al The Dfm1 Derlin Is Required for ERAD Retrotranslocation of Integral Membrane Proteins. Molecular Cell. 2018;69(2):306–320. 10.1016/j.molcel.2017.12.012 29351849 PMC6049073

[17] Ng W, Sergeyenko T, Zeng N, Brown JD, Romisch K. Characterization of the proteasome interaction with the Sec61 channel in the endoplasmic reticulum. Journal of Cell Science. 2007;120(4):682–91. 17264153 10.1242/jcs.03351

[18] Carvalho P, Goder V, Rapoport TA. Distinct Ubiquitin-Ligase Complexes Define Convergent Pathways for the Degradation of ER Proteins. Cell. 2006;126(2):361–73. 10.1016/j.cell.2006.05.043 16873066

[19] Schäfer A, Wolf DH. Sec61p is part of the endoplasmic reticulum-associated degradation machinery. The EMBO Journal. 2009;28(19):2874–84. 10.1038/emboj.2009.231 19696741 PMC2760108

[20] Schäuble N, Lang S, Jung M, Cappel S, Schorr S, Ulucan Ö, et al BiP-mediated closing of the Sec61 channel limits Ca2 leakage from the ER. The EMBO Journal. 2012;31(15):3282–96. 10.1038/emboj.2012.189 22796945 PMC3411083

[21] Stirling CJ, Rothblatt J, Hosobuchi M, Deshaies R, Schekman R. Protein translocation mutants defective in the insertion of integral membrane proteins into the endoplasmic reticulum. Molecular Biology of the Cell. 1992;3(2):129–42. 10.1091/mbc.3.2.129 1550957 PMC275513

[22] Shamu CE, Walter P. Oligomerization and phosphorylation of the Ire1p kinase during intracellular signaling from the endoplasmic reticulum to the nucleus. The EMBO Journal. 1996;15(12):3028–39. 8670804 PMC450244

[23] Sikorski RS, Hieter P. A system of shuttle vectors and yeast host strains designed for efficient manipulation of DNA in Saccharomyces cerevisiae. Genetics. 1989 5; 122(1):19–27. 2659436 10.1093/genetics/122.1.19 PMC1203683

[24] Plemper RK, Bordallo J, Deak PM, Taxis C, Hitt R, Wolf DH. Genetic interactions of Hrd3 and Der3/Hrd1 with Sec61 suggest a retro-translocation complex mediating protein transport for ER degradation. J Cell Sci. 1999; 112:4123–34. 10547371 10.1242/jcs.112.22.4123

[25] Stein A, Ruggiano A, Carvalho P, Rapoport TA. Key Steps in ERAD of Luminal ER Proteins Reconstituted with Purified Components. Cell. 2014;158(6):1375–88. 10.1016/j.cell.2014.07.050 25215493 PMC4163015

[26] Gillece P, Luz JM, Lennarz WJ, Cruz FJDL, Römisch K. Export of a Cysteine-Free Misfolded Secretory Protein from the Endoplasmic Reticulum for Degradation Requires Interaction with Protein Disulfide Isomerase. The Journal of Cell Biology. 1999;147(7):1443–56. 10613903 10.1083/jcb.147.7.1443 PMC2174254

[27] Vashist S, Ng DT. Misfolded proteins are sorted by a sequential checkpoint mechanism of ER quality control. The Journal of Cell Biology. 2004 12;165(1):41–52. 10.1083/jcb.200309132 15078901 PMC2172089

[28] Janke C, Magiera MM, Rathfelder N, Taxis C, Reber S, Maekawa H, et al A versatile toolbox for PCR-based tagging of yeast genes: new fluorescent proteins, more markers and promoter substitution cassettes. Yeast. 2004;21(11):947–62. 10.1002/yea.1142 15334558

[29] Walter P, Blobel G. Translocation of proteins across the endoplasmic reticulum. I. Signal recognition protein (SRP) binds to in-vitro-assembled polysomes synthesizing secretory protein. The Journal of Cell Biology. 1981 1;91(2):545–50. 7309795 10.1083/jcb.91.2.545 PMC2111968

[30] Savitski MM, Reinhard FBM, Franken H, Werner T, Savitski MF, Eberhard D, et al Tracking cancer drugs in living cells by thermal profiling of the proteome. Science. 2014 2;346(6205):1255784–. 10.1126/science.1255784 25278616

[31] Cox J, Mann M. MaxQuant enables high peptide identification rates, individualized p.p.b.-range mass accuracies and proteome-wide protein quantification. Nature Biotechnology. 2008;26(12):1367–72. 10.1038/nbt.1511 19029910

[32] Schwanhäusser B, Busse D, Li N, Dittmar G, Schuchhardt J, Wolf J, et al Global quantification of mammalian gene expression control. Nature. 2011;473(7347):337–42. 10.1038/nature10098 21593866

[33] Leitner A, Walzthoeni T, Aebersold R. Lysine-specific chemical cross-linking of protein complexes and identification of cross-linking sites using LC-MS/MS and the xQuest/xProphet software pipeline. Nature Protocols. 2013;9(1):120–37. 10.1038/nprot.2013.168 24356771

[34] Ritchie ME, Phipson B, Wu D, Hu Y, Law CW, Shi W, et al limma powers differential expression analyses for RNA-sequencing and microarray studies. Nucleic Acids Research. 2015;43(7). 10.1093/nar/gkv007 PMC4402510 25605792

[35] Huber W, Heydebreck AV, Sultmann H, Poustka A, Vingron M. Variance stabilization applied to microarray data calibration and to the quantification of differential expression. Bioinformatics. 2002 1;18(Suppl 1). 10.1093/bioinformatics/18.suppl_1.s96 12169536

[36] Gatto L, Lilley KS. MSnbase-an R/Bioconductor package for isobaric tagged mass spectrometry data visualization, processing and quantitation. Bioinformatics. 2011;28(2):288–9. 10.1093/bioinformatics/btr645 22113085

[37] Aiyar A, Xiang Y, Leis J. Site-Directed Mutagenesis Using Overlap Extension PCR. In Vitro Mutagenesis Protocols.:177–92. 10.1385/0-89603-332-5:177 8850005

[38] Horton RM, Hunt HD, Ho SN, Pullen JK, Pease LR. Engineering hybrid genes without the use of restriction enzymes: gene splicing by overlap extension. Gene. 1989;77(1):61–68. 2744488 10.1016/0378-1119(89)90359-4

[39] Servas C, Römisch K. The Sec63p J-Domain Is Required for ERAD of Soluble Proteins in Yeast. PLoS ONE. 2013 4;8(12). 10.1371/journal.pone.0082058 PMC3852996 24324744

[40] Esnault Y, Blondel M, Deshaies R, Scheckman R, Képès F. The yeast SSS1 gene is essential for secretory protein translocation and encodes a conserved protein of the endoplasmic reticulum. The EMBO Journal. 1993;12(11):4083–93. 8223425 10.1002/j.1460-2075.1993.tb06092.x PMC413701

[41] Foresti O, Rodriguez-Vaello V, Funaya C, Carvalho P. Quality control of inner nuclear membrane proteins by the Asi complex. Science. 2014;346(6210):751–5. 10.1126/science.1255638 25236469

[42] Vembar SS, Brodsky JL. One step at a time: endoplasmic reticulum-associated degradation. Nature Reviews Molecular Cell Biology. 2008 12;9(12):944–57. 10.1038/nrm2546 19002207 PMC2654601

[43] Baker RT, Tobias JW, Varshavsky A. Ubiquitin-specific proteases of Saccharomyces cerevisiae. Cloning of UBP2 and UBP3, and functional analysis of the UBP gene family. J Biol Chem. 1992 11; 267(32):23364–75. 1429680

[44] Christianson JC, Olzmann JA, Shaler TA, Sowa ME, Bennett EJ, Richter CM, et al Defining human ERAD networks through an integrative mapping strategy. Nature Cell Biology. 2011;14(1):93–105. 10.1038/ncb2383 22119785 PMC3250479

[45] Grubb S, Guo L, Fisher EA, Brodsky JL. Protein disulfide isomerases contribute differentially to the endoplasmic reticulum–associated degradation of apolipoprotein B and other substrates. Molecular Biology of the Cell. 2012;23(4):520–32. 10.1091/mbc.E11-08-0704 22190736 PMC3279382

[46] Vitu E, Gross E, Greenblatt HM, Sevier CS, Kaiser CA, Fass D. Yeast Mpd1p Reveals the Structural Diversity of the Protein Disulfide Isomerase Family. Journal of Molecular Biology. 2008;384(3):631–40. 10.1016/j.jmb.2008.09.052 18845159

[47] Voorhees R, Hegde R. The structure of the mammalian Sec61 channel opened by a signal sequence. 2016; 10.1126/science.aad4992 PMC4700591 26721998

[48] Kulak NA, Pichler G, Paron I, Nagaraj N, Mann M. Minimal, encapsulated proteomic-sample processing applied to copy-number estimation in eukaryotic cells. Nature Methods. 2014 2;11(3):319–24. 10.1038/nmeth.2834 24487582

