# Supplementary material for: Effect of Sec61 interaction with Mpd1 on endoplasmic reticulum-associated degradation
Source: PLoS One. 2019 Jan 25;14(1):e0211180. doi: 10.1371/journal.pone.0211180 (PMC6347170; doi:10.1371/journal.pone.0211180)
Supplement: S1 File — (DOC) [file pone.0211180.s002.doc]

Supplemental Methods

RNA isolation and HAC splicing PCR

For the isolation of RNA all solutions were RNAse free. Strains to be evaluated were grown to an OD_600_=1, and two 10 ml replicas per strain were made. To one replica tunicamycin (2 µg/ml of) was added, to the other DMSO (same volume as tunicamycin), and cells were grown for 3h more. Cells were then harvest at 4,500 x g for 5 min (4ºC), resuspended in 1 ml ice-cold DEPC-water, and transferred to an RNase-free tube. After sendimentation (13,000 x g, 10 sec, 4ºC) pellet was resuspended in 400 µl TES Solution (10 mM Tris-HCl, pH 7.7, 10 mM EDTA, 0.5% (w/v) SDS), 400 µl of Roti-Aqua-Phenol (Carl Roth) were added, and after vortexing (10 sec), samples were incubated for 1 h at 65ºC with occasional vortexing. Samples were then placed on ice for 5 min and centrifuged at 13,000 x g for 5 min (4ºC). Aqueous phase was transferred to a clean tube and 400 µl of Roti-Aqua-Phenol were added. Samples were vortexed for 20 sec and incubated for 5 min on ice. Samples were then centrifuged as before, aqueous phase transferred again to a clean tube, and 400 µl of chloroform were added. Samples were vortexed again (20sec) and sendimented (13,000 x g, 5 min, 4ºC). Aqueous phase was once more transferred to a clean tube, and 40 µl of 3M NaAc, followed by 1 ml of ice cold 100% ethanol, were added. After repeating the vortexing and sedimentation steps, pellets were washed with 1.5 ml of 70% ethanol and sedimented as before. Finally, samples were resuspended in 50 µL of DEPC-water and RNA concentration was determined using a NanoDrop spectrophotometer (ThermoFisher).

To generate cDNA from each RNA samples, the RNA samples were diluted to a concentration of 0.1 µg/ml and reverse-transcription reactions were made as follows using MaximaRT (ThermoFisher):

| Component | Volume (μl) | Final concentration |
| --- | --- | --- |
| RNA | 1 | 0.1 µg |
| Oligo(dT18)-primer (100 mM) | 1 | 100 pmol |
| dNTP mix (10 mM) | 1 | 0.5 mM |
| RNase-free dH2O | To 14.5 | to 14.5 µl |
| 5X RT buffer | 4 | 1x |
| RNasin (40 U/μl) | 0.5 | 20 U |
| Maxima RT | 1 | 200 U |

Samples were then incubated for 30 min at 50ºC followed by an inactivation at 85ºC for 5 min.

We then used 1 µl of each cDNA for PCR, using both the *HAC1*- (5’-CTGGCTGACCACGAAGAC and 5’- TTGTCTTCATGAAGTGATGGC-3’) and the *ACT1*- (5’-ATTCTGAGGTTGCTGCTTT-3’ and 5’- GTGGTGAACGATAGATGG-3’) specific primers.

Amplification reactions were done using KAPAHiFi Hot Start DNA (PEQLAB) and the program used was the following:

| Cycles | Step | Temperature | Duration |
| --- | --- | --- | --- |
| 1 | Initial denaturation | 95 | 5 sec |
| 35 | Denaturation | 98 | 20 sec |
|  | Annealing | 54 | 15 sec |
|  | Extension | 72 | 30 sec/kb |
| 1 | Final Extension | 72 | 5 min |
|  | Store | 4 | ∞ |

After PCR 10 µl of each reaction was resolved in an 1% agarose gel at 100V for 1h. Signal was acquired with the E-BOX VX2 gel documentation system (PEQLAB).
