# Supplementary figures and images for: Effect of Sec61 interaction with Mpd1 on endoplasmic reticulum-associated degradation
Source: PLoS One. 2019 Jan 25;14(1):e0211180. doi: 10.1371/journal.pone.0211180 (PMC6347170; doi:10.1371/journal.pone.0211180)

Supplementary Figure 1

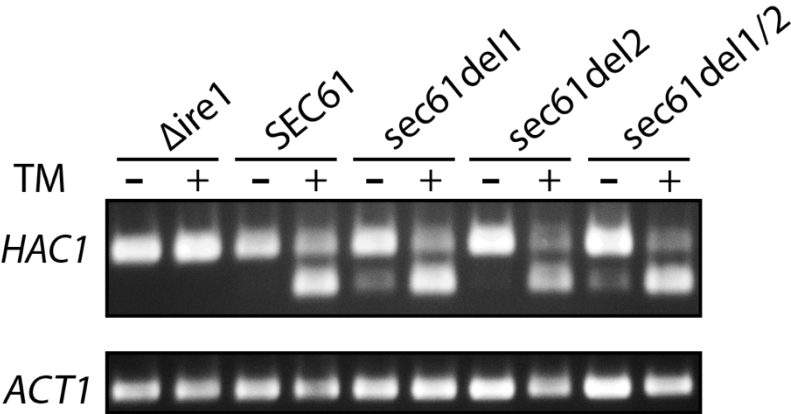

Supplement: S1 Fig — Wildtype and Sec61 hinge mutants were either treated with tunicamycin (2 μg/ml) (TM) or DMSO (control), followed by total RNA isolation, and cDNA production from isolated RNA. A quantitative PCR was done from equal amounts of cDNA. Agarose gel showing the resultant PCR products. Upper slice shows HAC1 PCR product. Upper bands (720 bp) represent the unspliced (uninduced) HAC1 mRNA, while lower bands (470 bp) represent the spliced (induced) HAC1 mRNA. Bottom slice show the actin PCR product. The Δire1 mutant was used as negative control. (PDF) [file pone.0211180.s003.pdf]

## Supplementary Figure 2

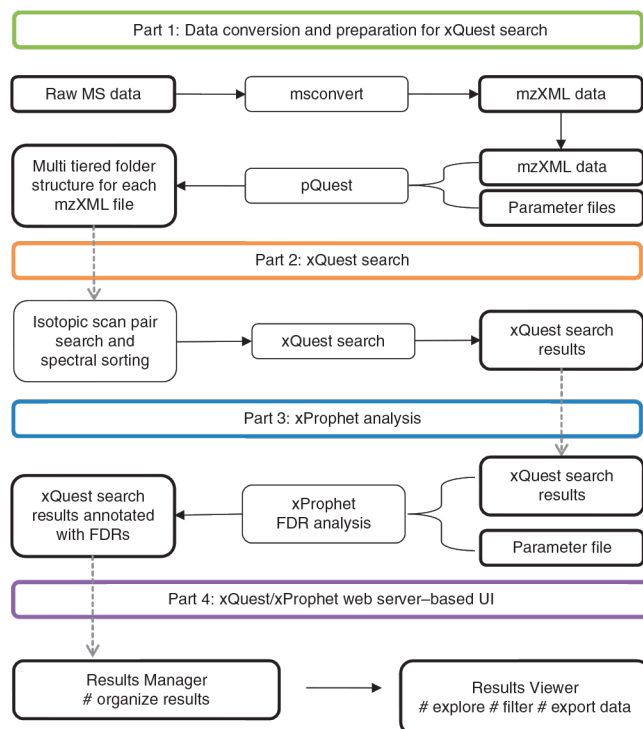

Supplement: S2 Fig — Workflow of the xQuest/xProphet software pipeline for the identification and statistical validation of cross-linked peptides from XL-MS experiments. The first step includes the conversion of raw MS data to the mzXML format and the preparation of the folder structure for the xQuest search. The second step includes the xQuest search and the identification of cross-linked peptides. The third step describes the statistical validation of the xQuest search results by xProphet, and the fourth step illustrates the web server–based data and result visualization. UI, user interface. (PDF) [file pone.0211180.s004.pdf]

Supplementary Figure 4

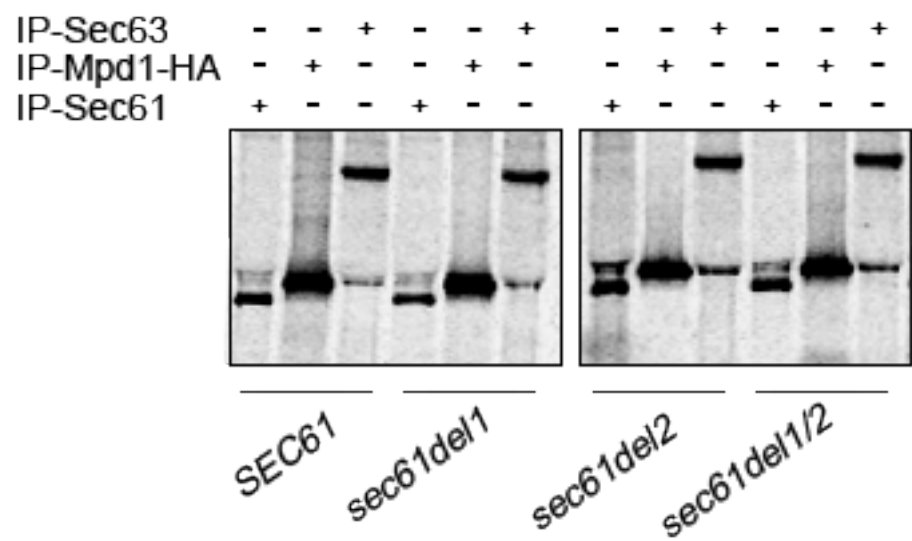

Supplement: S4 Fig — Samples were immunoprecipitated using saturating amounts of anti-Sec61 N-terminus, anti-HA, or anti-Sec63 antibodies. Conditions used for immunoprecipitation were the same as for the first immunoprecipitation done for Mpd1xSec61 interaction determination (Fig 4E) as well as in the same backgrounds. Samples were resolved by SDS-Page and signal acquired by phosphorimaging. (PDF) [file pone.0211180.s006.pdf]
