# Supplementary material for: Effect of Sec61 interaction with Mpd1 on endoplasmic reticulum-associated degradation
Source: PLoS One. 2019 Jan 25;14(1):e0211180. doi: 10.1371/journal.pone.0211180 (PMC6347170; doi:10.1371/journal.pone.0211180)

Supplementary Figure 3

A)

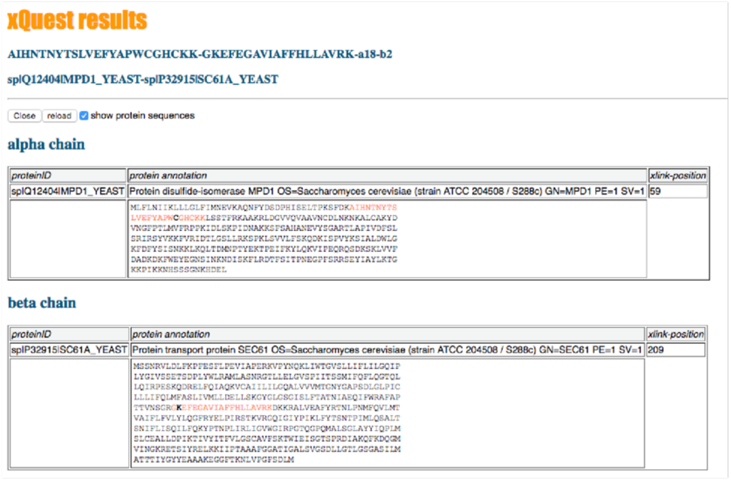

B)

	Sec61	Asi3	Mpd1	Sec63	YkH7	YN8B	YNS1
Cytosolic	K26	x		x	x		
Lumenal	K209	x	x				
	K229				x		
Cytosolic	K273						x
	K393				x		
	K396	x		x	x		x
	K405	x		x	x	x	x
	K470			x	x	x	x

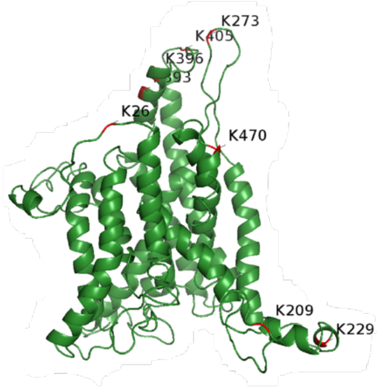

Supplement: S3 Fig — Example of the returned results after xQuest/xProphet analysis. A) Detected Sec61xMpd1 crosslinked site. B) Resume of the detected crosslinked sites detected by the software in a given analysis. A mapping of the detected crosslinked positions onto Sec61 can also be seen. (PDF) [file pone.0211180.s005.pdf]
